# Concise Review: Bone Marrow for the Treatment of Spinal Cord Injury: Mechanisms and Clinical Applications

**DOI:** 10.1002/stem.570

**Published:** 2010-11-23

**Authors:** Karina T Wright, Wagih El Masri, Aheed Osman, Joy Chowdhury, William E B Johnson

**Affiliations:** a Spinal Studies and Midlands Centre for Spinal Injuries, RJAH Orthopaedic HospitalOswestry, Shropshire, United Kingdom; b Institute of Science and Technology in Medicine, Keele UniversityKeele, Staffordshire, United Kingdom; c School of Life and Health Sciences, Aston UniversityBirmingham, United Kingdom

**Keywords:** Adult human bone marrow, Spinal cord injury, Cell transplantation, Clinical translations and clinical trials

## Abstract

Transplantation of bone marrow stem cells into spinal cord lesions enhances axonal regeneration and promotes functional recovery in animal studies. There are two types of adult bone marrow stem cell; hematopoietic stem cells (HSCs), and mesenchymal stem cells (MSCs). The mechanisms by which HSCs and MSCs might promote spinal cord repair following transplantation have been extensively investigated. The objective of this review is to discuss these mechanisms; we briefly consider the controversial topic of HSC and MSC transdifferentiation into central nervous system cells but focus on the neurotrophic, tissue sparing, and reparative action of MSC grafts in the context of the spinal cord injury (SCI) milieu. We then discuss some of the specific issues related to the translation of HSC and MSC therapies for patients with SCI and present a comprehensive critique of the current bone marrow cell clinical trials for the treatment of SCI to date. Stem Cells 2011;29:169–178

## SPINAL CORD INJURY AND THE INTRINSIC RESPONSE

When axons in the central nervous system (CNS) are damaged they mount a poor regenerative response due to a combination of inflammation, resulting in extensive neuronal and glial cell death and glial cell activation and hypertrophy, which contributes to the formation of the glial scar. These intrinsic responses to tissue injury both contribute to an environment that is inhibitory to axonal regrowth [1].

### Inflammation

Following spinal cord injury (SCI), the blood-brain barrier is disrupted and an influx of inflammatory cells occurs, which is facilitated by their expression of matrix metalloproteinases (MMPs) [2]. MMPs, other proteolytic and oxidative enzymes, and proinflammatory cytokines that are produced by infiltrating neutrophils and macrophages, along with resident microglia, induce a reactive process of secondary cell death in the tissue that surrounds the original injury site [2–4]. This secondary damage continues in the days and weeks following SCI, which may lead to an increase in cavitation and cyst formation at the center of the lesion, exacerbating neurological dysfunction [5].

Some evidence suggests that inflammation may be a beneficial response to SCI. For example, macrophages phagocytose the myelin debris present in the injured spinal cord, which is known to inhibit axonal regeneration [6, 7], and increase in the number of macrophages in a CNS injury can promote nerve regrowth [8]. In addition, macrophages may also release protective cytokines such as basic fibroblast growth factor, nerve growth factor (NGF), and neurotrophin 3, which promote neuronal regeneration and tissue repair [9].

### Glial Scarring

Glial scarring involves astrocytes, which are activated in an effort to restore the blood-brain barrier, and oligodendrocytes. The extracellular matrix produced by these scar-associated cells contains a number of molecules that inhibit axonal regrowth [10] of which chondroitin-sulfated (CS) proteoglycans (PG) are the major inhibitory molecules synthesized by reactive astrocytes. CSPGs consist of a protein core to which glycosaminoglycan (GAG) side chains are attached. Much of the evidence suggests that the inhibitory activity of CSPGs is derived from their CS GAG side chains, as treatments with chondroitinase ABC (which cleaves these chains) reduces CSPG inhibition to neurites in vitro [11] and regenerating axons in vivo [12].

Other inhibitory molecules present within the glial scar include myelin-associated proteins, such as myelin-associated glycoprotein (MAG), Nogo-A, and oligodendrocyte-myelin glycoprotein (OMgp) [6, 7]. MAG is a potent inhibitor of neurite outgrowth when used as a culture substrate [6], which is expressed by oligodendrocytes and Schwann cells. MAG signals through the Nogo-66 receptor complex (NgR), but there are several other neuronal receptors, which interact with the NgR complex and MAG to influence downstream signaling [13]. Nogo-A and OMgp are also derived from oligodendrocytes and act as inhibitors of axonal growth [14]. A number of different regions of Nogo-A contribute to its inhibitory activity, and it is probable that these different regions bind to not only the NgR complex but also to unidentified Nogo-A receptors in the CNS [14]. In contrast, OMgp appears to be dependent on the NgR complex, as cleavage of NgR renders axons insensitive to OMgp-induced growth inhibition [15].

## HOW MIGHT BONE MARROW STEM CELL TRANSPLANTATION HELP HEAL THE INJURED SPINAL CORD?

There are two types of bone marrow stem cell, hematopoietic stem cells (HSCs) and mesenchymal stem cells (MSCs), which are known to differentiate into hematopoietic and mesenchymal cell lineages, respectively (supporting information Fig. 1). For clinical transplantation, HSCs and MSCs represent attractive cell sources as they can be easily and reproducibly isolated from bone marrow aspirates and reintroduced into patients as autografts. In animal models of SCI, their transplantation has promoted remyelination [16–18], axonal sparing, and functional recovery [19–31]. Many studies have documented successful engraftment of HSCs and MSCs into the injured spinal cord [19–31].

### HSC and MSC Isolation, Culture, and Characterization

HSCs are defined by their lifelong ability to reconstitute all of the hematopoietic lineages in transplanted hosts [32]. Although HSCs have been shown to proliferate in vivo, there are as yet no definitive in vitro assays to detect and expand purified HSCs, as HSCs in long-term culture form progenitor populations that differentiate along the hematopoietic lineages. Researchers have yet to find a single molecular marker that is exclusively expressed by HSCs. However, HSCs can be distinguished and isolated from mature blood cells by their lack of lineage-specific markers and presence of other cell surface antigens such as CD34 and CD133 [33]. CD34 has been used routinely to enrich freshly isolated hematopoietic cell populations, which include HSC, for clinical transplantation in patients [34]. MSCs are a population of cells that differentiate along various mesenchymal lineages, for example, to form osteoblasts, adipocytes, and chondrocytes [35]. These multipotent cells have received considerable interest as possible donor cells for cell transplantation therapies because MSCs can be isolated from bone marrow with relative ease. Adherent stromal cells (MSCs) will outgrow any fully differentiated and nonproliferating cells, which might also adhere to bone marrow mononuclear cell seeded-culture plates. Unlike HSCs, MSCs can be culture expanded to generate large numbers [36]. Similar to HSCs, a single molecular marker that is exclusively expressed by MSCs is yet to be found, although the International Society for Cellular Therapy has stated that MSCs must express CD105, CD73, and CD90 and lack expression of CD45, CD34, CD14, CD11b, CD79a, or CD19, and human leukocyte antigen-DR (HLA-DR) surface molecules [37].

### HSCs and MSCs As Replacements for Lost Glial Cells and Neurons

Some evidence has suggested that HSCs and MSCs may transdifferentiate along glial and neuronal pathways [23, 27, 38–41]. The topic of MSC neural transdifferentiation in particular has been extensively reviewed elsewhere [38–41]. In brief, many of these studies have reported that HSCs and MSCs have the ability to form cells of a glial and neuronal lineage in response to various types of genetic, chemical, and/or physiological induction. In most cases, the characterization of cell phenotype was limited to the detection of lineage-specific markers with no glial or neuronal cell function apparent, that is, myelin synthesis or electrophysiological activity. There is some controversy regarding the capacity of MSCs to transdifferentiate into neural cells in vitro and in vivo. The differentiation of stem cells toward a neuronal lineage in development is a complex and gradual progression. In contrast, in vitro studies have described neuronal differentiation in a matter of hours following the treatment of MSC with chemical agents (e.g., β mercaptoethanol, dimethyl sulfoxide, and butilated hydroxyanisole), which is highly questionable. Such chemically induced transdifferentiation of various cell types including primary rat fibroblasts, rat PC-12 cells (a cell line that is used to model neuronal differentiation), and MSCs has previously been tested [42]. On application of induction medium, all cell types altered morphologically and appeared to possess fine neurite-like extensions. However, time lapse analysis indicated that these structures were due to cellular shrinkage and not to neurite extension proper. These researchers went on to introduce various other known cell stressors, including detergents, sodium chloride, and extreme pH levels, which also produced a similar morphological change to give the appearance of neuronal differentiation. Cellular shrinkage could also explain the apparent increase in immunoreactivity of neuronal markers (e.g., β III tubulin) exhibited in these differentiation protocols, as immunolocalization in cells, which had retracted cell processes, would appear to be more intense than in spread cells, which had received no treatment [42]. Doubts were also raised regarding the interpretation of in vivo studies that have reported transdifferentiation of MSCs [43, 44], where it has been suggested that the supposed MSC differentiation into neuronal phenotypes were rather a result of fusion between donor MSCs and host neural cells, which lead to false immunopositive characterization [45]. However, some studies have demonstrated phenotypic functions in transplanted HSC and MSC, that is, nerve myelination and electrophysiological activity for evident glial and neuronal phenotypic function [27, 46–48]. Interestingly, neuronal induction of MSCs prior to their transplantation into SCI lesions was not necessary to promote axonal regeneration when induced and noninduced MSC grafts were directly compared [49]. In addition, the glial or neuronal differentiation of HSCs and MSCs prior to their transplantation into CNS injury sites was not necessary to promote the remyelination, axonal regeneration, and functional recovery noted by the majority of investigators in the field [16–31]. Therefore, there is a clear possibility that HSCs and MSCs may have beneficial effects that extend beyond their potential to differentiate in vitro to form replacement cells of a glial or neuronal lineage.

### MSCs Can Modify the SCI Milieu to Support Axonal Regeneration

The precise mechanisms by which transplantation of HSCs and MSCs promote functional recovery after SCI is still unclear. HSCs secrete some neurotrophic growth factors, such as angiopoietin-1 and have been suggested to encourage vascularization [50] and hence encourage wound healing in SCI. However, the majority of data available describes how MSC grafts can influence the SCI milieu, and therefore, this review has focused on MSC mechanisms (supporting information Fig. 2). There is increasing evidence that MSCs may be immunosuppressive [51–54]. These immunosuppressive properties may combine to reduce the acute inflammatory response to SCI and hence reduce cavity formation as well as decrease astrocyte and microglia/macrophage reactivity [26, 30, 55]. MSC transplantation has been shown not only to enhance tissue preservation after SCI but also to associate with a reduction in injury-induced sensitivity to mechanical stimuli in an experimental SCI model, which is functionally indicative of anti-inflammatory activity [55]. Overall, these findings indicate that MSC transplantation into SCI lesions attenuates acute inflammation and that this is beneficial to the recovery of function following SCI. However, SCI initiates an innate immune response that participates not only in secondary pathogenesis but also in wound healing [56], therefore further research into the use of MSC as modulators of the immune system is required.

Transplanted MSCs might bring about CNS functional recovery by modifying the SCI milieu directly. MSCs may promote axonal regeneration or encourage functional plasticity by establishing an environment, which supports axonal growth, for example, by abrogating the inhibitory influence of the glial scar. MSCs synthesize a number of neurotrophic cytokines that stimulate nerve growth, including brain-derived neurotrophic factor, NGF, and vascular endothelial growth factor (VEGF) [26, 57], and we, and others, have shown that MSC conditioned media (MSC CM) stimulates neurite outgrowth in vitro [26, 58]. However, we have also demonstrated that the stimulus of MSC CM was insufficient to promote nerve growth over inhibitory molecules that are present in the glial scar, that is, CSPGs, MAG, and Nogo-A [56]. An important interpretation of this finding is that the neurotrophic factors secreted by MSCs may have limited effect in the context of the SCI milieu.

It has been proposed that MSCs act as “guiding strands” for regenerating axons across the lesion site in the injured cord and along spinal cord tracts in vivo [20]. Transplanted MSCs were seen to form bundles that bridged the lesion, which were also populated with immature astrocytes and nerve fiber outgrowths [20]. In coculture experiments, we used time lapse microscopy to demonstrate that MSCs can act directly both to provide contact guidance and cellular bridges over nerve-inhibitory matrices [58]. Human MSCs express various cell adhesion molecules and receptors [57] that may function in MSC: neuronal interactions and hence axonal regeneration. These include ninjurin 1 and 2, Netrin 4, neuronal cell adhesion molecule [57], Robo1, and Robo4, which are all known to regulate neuronal cell migration and axon guidance in development [59]. Alternatively, MSCs might degrade nerve-inhibitory molecules present in the SCI milieu. Human MSCs express membrane type I matrix metalloproteinase and MMP2, which degrade CSPGs [60–62]. Another interesting possibility is that transplanted MSCs synthesize nerve-permissive matrix components within the lesion that may contribute to the decrease in cavitation noted in some studies [21, 22], for example, laminin, fibronectin, and collagen [22]. Evidence that MSCs provide a supportive environment for neurite elongation has been shown in vitro, where a feeder layer of MSC enhanced the development of neural networks from neurospheres isolated from fetal rat spinal cords [21].

A recent study has focused on the ability of MSCs to respond to the environmental stimuli in the injured spinal cord. MSCs that were administered with extracts from injured spinal cord tissue responded by increasing their synthesis of various cytokines, including IL-6, IL-7, and VEGF [63]. The biological significance of the elevated secretion of these cytokines is difficult to interpret as each factor could play a functional role in wound repair as well as a detrimental role in secondary tissue damage. However, this study demonstrated that there was a dynamic relationship between the transplanted MSCs and the host SCI environment. Elucidating and manipulating these interactions will provide an extremely complex area for future scientific research.

## THE TRANSLATION OF BONE MARROW CELL TRANSPLANTATION TO THE CLINIC

### HSC and MSC Populations in Humans with SCI

A preliminary question for the application of autologous HSCs or MSCs for human SCI cell therapy is whether these cells are available in individuals who have been injured. Early work demonstrated marked and significant changes in the composition of iliac crest tissue in individuals with complete paralysis compared with non-SCI donors [64]. In the 12–25 weeks after SCI, trabecular bone volume decreases by 30%, whereas the volume of bone marrow adipose tissue increases. The loss of mechanical loading following SCI is suggested to be a crucial stimulus for bone resorption [65]. However, surgical and chemical denervation in animal models leads to bone loss in both loaded and unloaded bones [66], which suggests that denervation in itself can contribute to the skeletal pathology observed following SCI. It is intuitive that such changes in the bone marrow microenvironment will have an impact on cells resident within marrow, although there is little data on whether this does occur. HSC populations are affected by SCI, where a reduced presence of long-term colony-forming dendritic cells has been determined. This loss of hematopoietic potential may have a role to play in the depressed natural and adaptive immunity seen in patients with SCI [67]. For MSCs, one study has reported successfully isolating “fibroblast-like mesenchymal cells” in just 75% of bone marrow aspirates tested from patients with SCI [68], which may suggest that the MSC population is also affected. However, more recently, we have shown that MSCs were generated from all SCI donor bone marrow samples that we have examined and that these MSCs were little if at all different to those isolated from non-SCI donors [69]. Importantly, we also found that MSCs from SCI donors were able to promote nerve growth, at least in vitro [69]. These findings bode well for the future development of bone marrow cell therapies for the treatment of SCI.

### Clinical Applications of Bone Marrow Cell: Cell Type and Number, Mode, and Time of Delivery

In practice, most clinical applications of bone marrow cells for the treatment of SCI have involved the use of whole mononuclear cells preparations (MCPs) [70–75] and two have used culture expanded MSCs [76, 77]. MCPs constitute hematopoietic cells of various stages of differentiation and endothelial cells as well HSCs and MSCs. No studies have directly compared the efficacy of these various bone marrow cell preparations in the clinic, although a direct comparison was recently made between human MCPs and culture expanded MSCs transplanted into a SCI model in rats, where no differences were reported with regard to graft efficiency, spinal cord tissue sparing, or glial scar reduction [78].

The issue of scaling up potential therapeutics is an area in SCI research that is not well documented but has important implications in the clinical setting when the lesion size in animal models and humans differ greatly. A typical injury in rat models of SCI is 1–3 mm in length into which, generally, 1–5 × 10^6^ cells are grafted (summarized in Table 1). In humans, it is perhaps intuitive to consider that more cells may be needed for larger lesions. In addition, if the acute stage of SCI proves a window of opportunity where grafting has beneficial effects then this large cell number must be generated rapidly. Seeding MSCs at low densities significantly reduces the MSC culture doubling time and greatly increased the overall MSC yield [69, 80], which has important implications clinically if MSC number is critical to the success of an MSC graft.

**Table 1:** Animal models of SCI and bone marrow stem cell transplantation

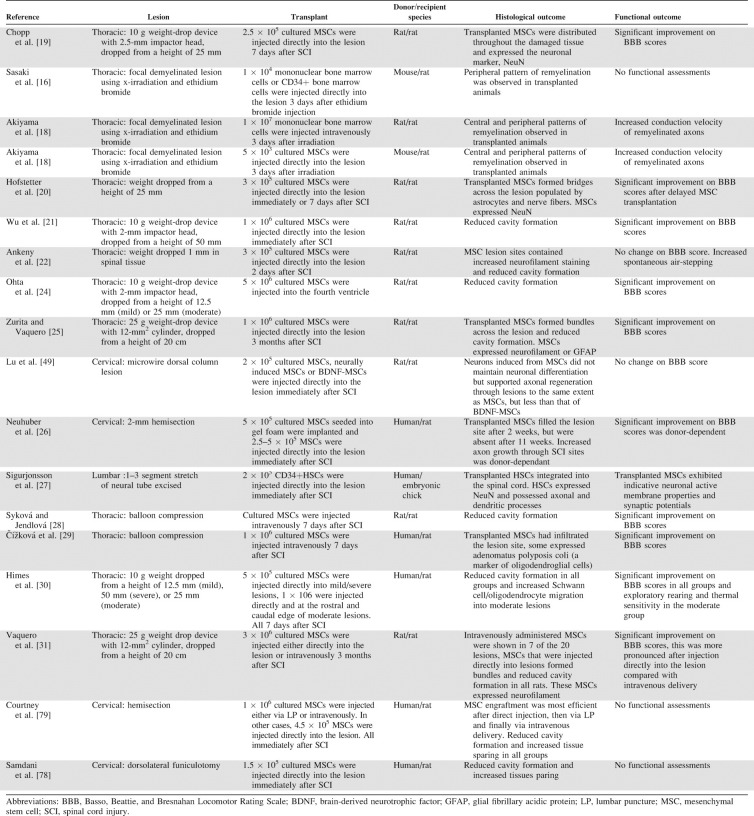

The delivery of HSCs and MSCs into animal CNS injury models varies considerably (Table 1). The method of cell delivery is of great importance to the clinic as injections directly into the spinal cord tissue may cause further damage. However, MSCs exhibit trophism for sites of tissue damage [81] and this may negate the need to inject cells directly into the injury site. Intravenous (IV) applications of MSCs in rodent models of SCI and brain trauma have shown that labeled MSCs can migrate toward and integrate into damaged CNS tissues up to 3 months post-transplantation [82]. MSCs have also been injected directly into the cerebrospinal fluid by lumbar puncture (LP) in animal models of SCI, where they migrated into injured spinal tissue and reduced cyst size and increased functional recovery [24, 79, 83, 84]. A direct comparison between the efficacy of these modes of delivery (IV vs. LP) and their effects on the host has previously been made [79]. In this study, human MSC engraftment into the injured spinal cord tissue in rats was determined as a percentage of total cord volume at 4 and 21 days after MSC delivery. When MSCs were injected intravenously, MSC engraftment was reported at 2.3% and 1.6%, whereas LP delivery increased MSC engraftment to 4.1% and 3.4% after 4 and 21 days, respectively. In addition, the increased engraftment of LP-delivered cells was associated with a decreased host immune response, increased tissue sparing, and decreased glial scarring compared with animal, which were injected intravenously [79]. This study highlights the importance of cell number in determining the outcome of cell transplantation; furthermore, the study represents a promising advance to the clinical use of MSC in SCI treatment as IV and LP colony-stimulating factor (CSF) infusion are minimally invasive delivery techniques.

The majority of HSC and MSC transplantations in animal models of SCI occur in the acute injury phase [19–24, 26–30]. However, there are a number of studies using chronic models of SCI in animals that have reported increased functional recovery following MSC transplantation 6–12 weeks after injuries were induced, which is considered chronic in these model systems [25, 31]. This literature indicates that both the acute, subacute, and chronic injury may well be a therapeutic target for MSC grafting. The acute or subacute milieu of the damaged spinal cord may influence the mechanism by which HSC or MSC graft might induce tissue protection/repair in a manner that differs to the chronic setting (e.g., in the acute setting for anti-inflammatory purposes or in the subacute/chronic setting for neurostimulatory and cell bridging effects and possibly glial or neuronal cell replacement). No physical therapy following HSC or MSC transplantation has been reported in any of the animal models reviewed in this article. It will be important to study these effects in future studies using HSCs and MSCs, as locomotor training activity when combined with other types of cell transplant has previously been reported to improve functional recovery in animal models of SCI [85].

### Current Bone Marrow Cell Clinical Trials for the Treatment of SCI

The current bone marrow cell clinical trials for the treatment of SCI are summarized in Table 2. There are no definitive rules for the classification of SCI as acute, subacute, or chronic. In general, provided there are no life-threatening-associated injuries or complications, the acute stage is likely to last up to the end of the period of spinal shock during which the patient is at the highest risk of developing complications. However, the presence of life-threatening-associated injuries or complications can prolong the acute stage until such conditions no longer pose a threat. The subacute stage can be described as the period during which all systems of the body that are affected by the SCI are managed and retrained to function as safely and as conveniently as possible. This usually lasts up to 6 months, occasionally longer. The International Campaign for Cures of SCI Paralysis (ICCP) have stated that “based on the available data, it might be suggested that the chronic state is only attained 12 months after SCI (where the preceding 6 months have indicated no change in functional capacity, thereby providing a stable baseline).” [86]. However, the criteria for acute, subacute, and chronic SCI are disputable and vary greatly among the clinics reviewed in this article. Therefore, we have described each trial according to their respective clinical classification, while also including the actual times of injury onset. In two of these studies, MCPs have been trialed in conjunction with granulocyte-macrophage colony-stimulating factor (GM-CSF) administration. GM-CSF has previously been shown to mobilize MCPs into the injured spinal cord and promote functional recovery from SCI in mice [87]. For these clinical trials, it was hypothesized that GM-CSF would not only promote the migration of MCPs into the lesioned spinal cord but also would have a direct effect on the transplanted cells by enhancing their survival and activating them to secrete neurotrophic cytokines [70, 73]. The first trial used a combination of MCPs with administration of GM-CSF in the acute setting, that is, within 7 days of injury with cells injected directly into the lesion site [70]. Of the six patients who were treated, five showed slightly improved neurological function. This same group of researchers have now gone on to treat a further 17 patients with SCI at 2 weeks postinjury (i.e., still acute), 6 patients between 14 days to 8 weeks postinjury (subacute), and 12 patients at >8 weeks postinjury (chronic) [73]. A control group of 13 patients were also included; these patients were treated only with conventional decompression and fusion surgery. In this latter study, 29.5% of the acute, 33.3% of the subacute, 0% of the chronic, and 7.7% of the control patients demonstrated an increase in neurological function at 10 months post-transplantation. However, as few patients have been treated at this stage, it is not clear whether the neurological improvements noted were directly attributable to the treatment and were not due to an intrinsic repair process and natural recovery.

**Table 2:** Clinical trials for the treatment of SCI using bone marrow

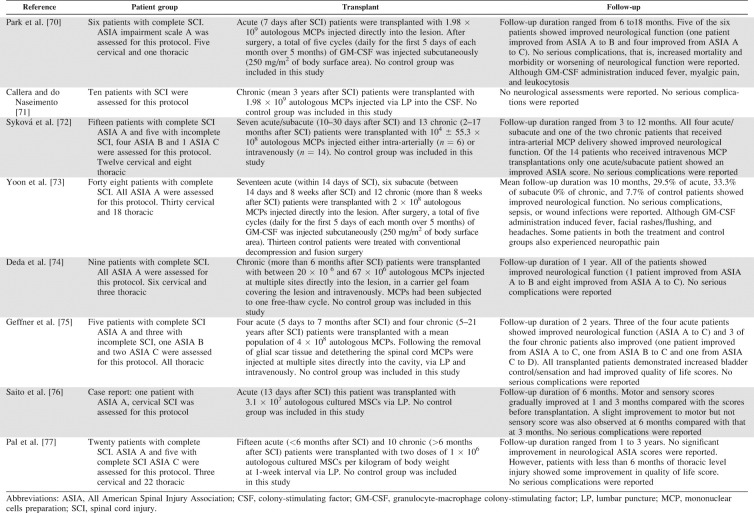

A preliminary safety study on the use of MCPs delivered via LP also with administration of GM-CSF for the treatment of SCI has been reported [71]. Ten patients with SCI were treated 4 hours after the bone marrow was aspirated and 100 million MCPs were injected. This brief study reported that no serious adverse effects were observed at 12 weeks follow-up, although no detailed neurological assessments were performed [71]. Another trial safely treated 20 patients with SCI ranging from 10 to 467 days postinjury with MCPs injected intra-arterially or by IV within 5 hours of harvesting [72]. The improved neurological outcome reported in one chronic patient who was neurologically stable for several months prior to cell implantation is promising. A case report on SCI treatment via LP delivery of cultured MSCs, where the patient was treated 13 days after SCI, reported that no adverse effects were noted in the 6 months follow-up to the treatment and both motor and sensory neurological scores gradually improved [76]. However, as with previous solely acute studies, these improvements are difficult to separate from an intrinsic repair process. Indeed, in a recent study using LP MSC transplantation for SCI repair in a more extensive cohort of patients, only the acute patient group demonstrated any improvement in quality of life score and patients with chronic injuries failed to show any improvements [77]. In contrast, increased functional recovery and improved quality of life was reported after treating four acute and four patients with chronic SCI with ∼800 million MCPs via multiple routes, ∼200 million cells were injected directly into the injury site after the removal of glial scar tissue and ∼300 million cells were delivered by both LP and IV administration [75]. Similar functional improvements have been reported in nine chronic patients following transplantation directly into the spinal cord tissue with MCPs, which had been subjected to a freeze-thaw cycle, suggesting that cryopreserved MCPs do not lose the ability to promote functional recovery [74]. Therefore, harvested cells could be cryopreserved and stored for future use. The improved neurological outcome reported in these chronic patients is exciting, although a control group was again not included in either study for comparison [74, 75]. Inclusion of a control group is of particular importance for the former study to access the effects of scar removal in the absence of MCP transplantation for comparison.

Larger patient cohorts would be required to determine the significance of any functional improvements in these patient trials and to assess any associated risks of MCP/MSC or GM-CSF treatments. It is noteworthy that no details of physical rehabilitation were reported in any of these clinical trials other than that “all patients underwent standard physical therapy prior to and after transplantation” [75]. For future reporting of clinical trials it will be important to include the details of any physical rehabilitation programs, which have previously been demonstrated to impact significantly on SCI recovery [88]. It is currently unclear whether cell transplantation in future SCI treatments should be limited to the acute, subacute, or chronic phase of injury. Indeed, it is highly likely that all of these stages may be targeted. However, when considering suitable patients to include for clinical trials, chronic patients with a stable neurological function (or dysfunction) would give the clearest indication that any functional improvements following bone marrow cell transplantation were due to that treatment and were not due to natural recovery [89]. To address these issues, the ICCP has published clear guidelines for devising future clinical trials (http://www.campaignforcure.org/iccp) [86]. This in itself is exciting news, which suggests that as a whole, the field of SCI research is an active area close to meaningful clinical translation.

## CONCLUSION

The potential of bone marrow cell transplantation as a method of repair in the injured CNS may serve a number of different purposes that span various therapeutic targets. Animal studies have demonstrated that transplanted MSCs modify the inflammatory environment in the acute setting and reduce the effects of the inhibitory scar tissue in the subacute/chronic setting to provide a permissive environment for axonal extension. In addition, grafted cells may provide a source of growth factors to enhance axonal elongation across spinal cord lesions. Other studies have suggested that HSCs and MSCs may even transdifferentiate to replace lost or damaged neuronal tissue. Preliminary clinical data indicates that autologous bone marrow cell transplantation and/or GM-CSF administration can be used to treat patients with SCI without any immediate serious complications. These data are promising, but future studies must continue to establish whether bone marrow cell treatments can serve as a safe and functional autologous source for the treatment of the injured CNS.
