# Supplementary figures and images for: Concise Review: Bone Marrow for the Treatment of Spinal Cord Injury: Mechanisms and Clinical Applications
Source: Stem Cells. 2010 Nov 23;29(2):169–78. doi: 10.1002/stem.570 (PMC3083520; doi:10.1002/stem.570)

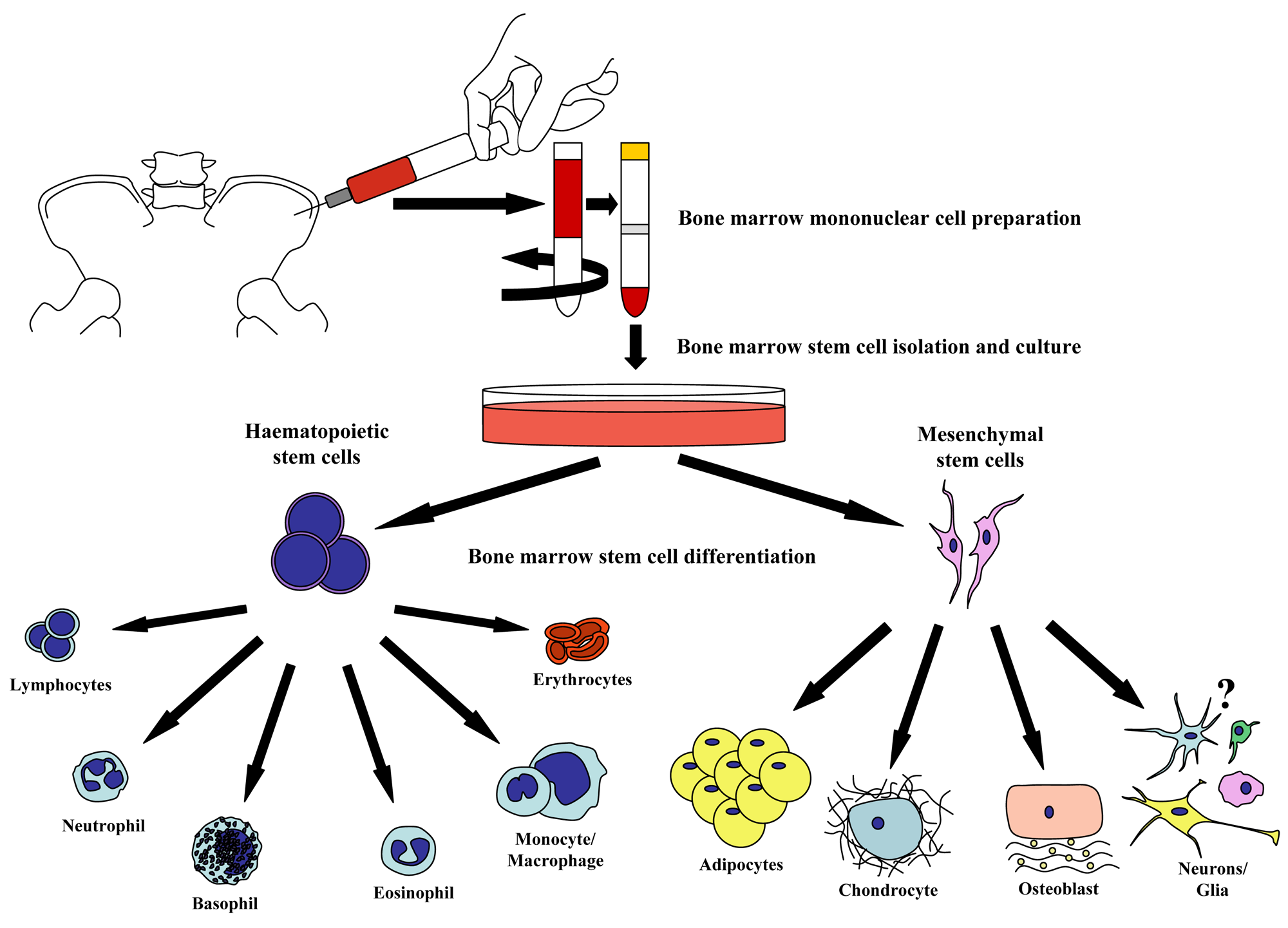

Supplement: Supplementary file 1 [file stem0029-0169-SD1.tif]

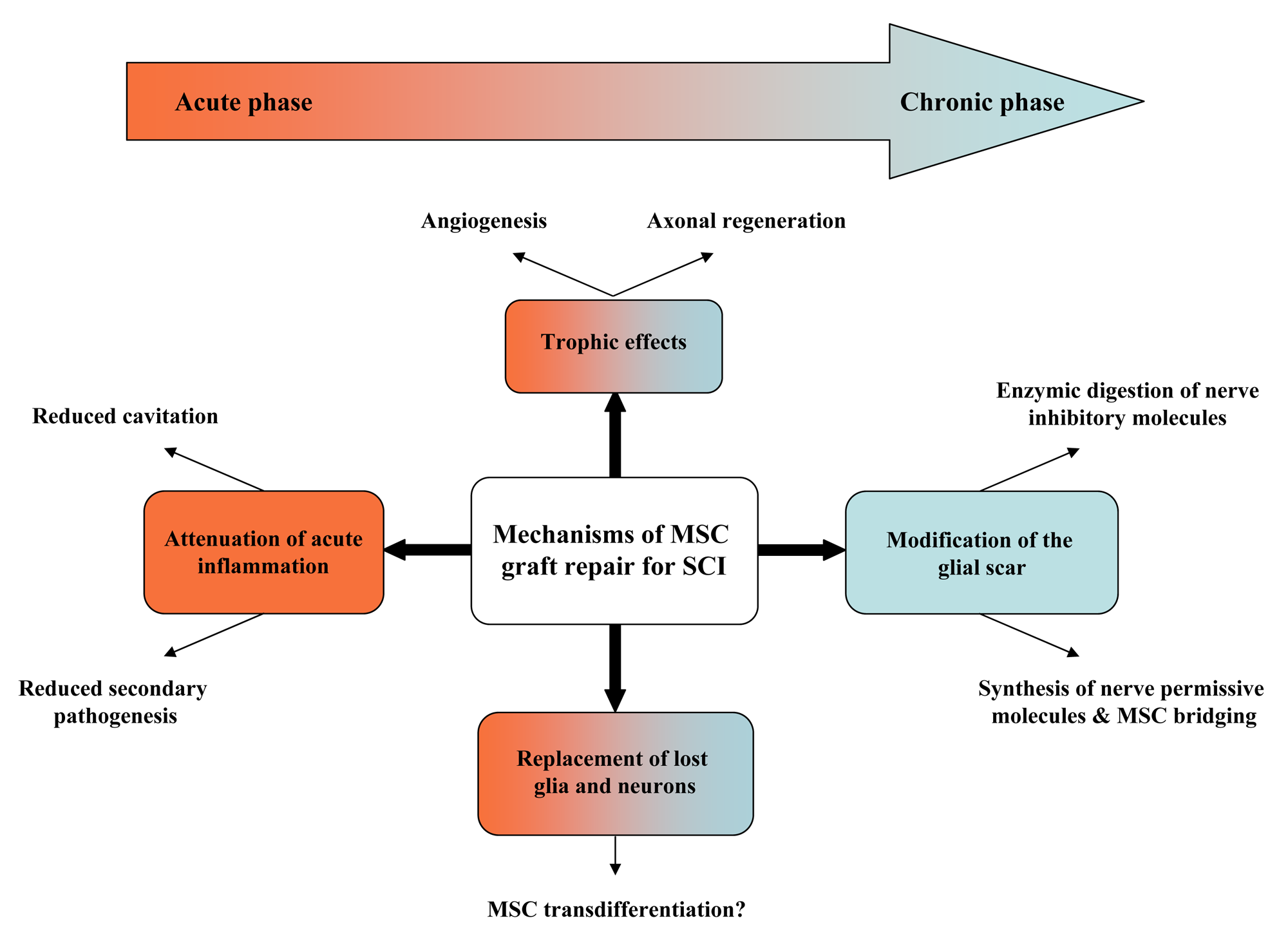

Supplement: Supplementary file 2 [file stem0029-0169-SD2.tif]
